# AGE DIFFERENCES IN CONCORDANCE BETWEEN LAB AND DAILY LIFE STRESS RESPONSES

**DOI:** 10.1093/geroni/igz038.2715

**Published:** 2019-11-08

**Authors:** Gloria Luong, Carla M Arredondo

**Affiliations:** Colorado State University, Fort Collins, Colorado, United States

## Abstract

The literature is mixed with respect to how stress reactivity changes with age. Previous studies have overlooked contexts, ignoring whether stressors occurred in the laboratory or in daily life. The Health and Daily Experiences (HEADE) study includes 126 younger and older adults who completed both laboratory stressors and ecological momentary assessments (EMA) of affect and stressor experiences in daily life. We found that the laboratory stressor elicited the greatest levels of negative affect reactivity (i.e., larger increases in negative affect) and positive affect reactivity (i.e., larger reductions in positive affect) compared to the two types of daily life stressors. Interpersonal stressors were associated with greater negative and positive affect reactivity compared to non-interpersonal stressors in daily life. Younger adults exhibited greater stress reactivity than older adults. Together, these findings support age-related reductions in stress reactivity. Implications for understanding stressor-health links are discussed.

